# Effect of Polymer Composition and Morphology on Mechanochemical Activation in Nanostructured Triblock Copolymers

**DOI:** 10.1021/acs.macromol.2c02475

**Published:** 2023-03-02

**Authors:** Zijian Huo, Swati Arora, Victoria A. Kong, Brandon J. Myrga, Antonia Statt, Jennifer E. Laaser

**Affiliations:** † Department of Chemistry, University of Pittsburgh, 219 Parkman Ave., Pittsburgh, Pennsylvania 15260, United States; ‡ Materials Science and Engineering, Grainger College of Engineering, University of Illinois, Urbana−Champaign, Illinois 61801, United States

## Abstract

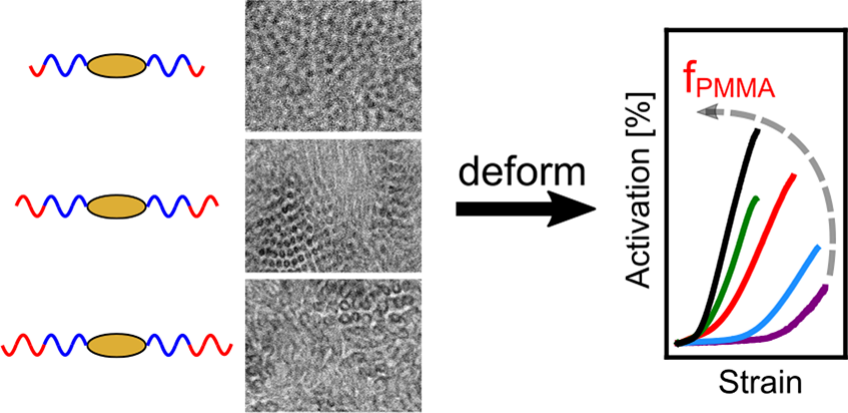

The effect of composition and morphology on mechanochemical activation in nanostructured block copolymers was investigated in a series of poly(methyl methacrylate)-*block*-poly(*n*-butyl acrylate)-*block*-poly(methyl methacrylate) (PMMA-*b*-PnBA-*b*-PMMA) triblock copolymers containing a force-responsive spiropyran unit in the center of the rubbery PnBA midblock. Triblock copolymers with identical PnBA midblocks and varying lengths of PMMA end-blocks were synthesized from a spiropyran-containing macroinitiatior via atom transfer radical polymerization, yielding polymers with volume fractions of PMMA ranging from 0.21 to 0.50. Characterization by transmission electron microscopy revealed that the polymers self-assembled into spherical and cylindrical nanostructures. Simultaneous tensile tests and optical measurements revealed that mechanochemical activation is strongly correlated to the chemical composition and morphologies of the triblock copolymers. As the glassy (PMMA) block content is increased, the overall activation increases, and the onset of activation occurs at lower strain but higher stress, which agrees with predictions from our previous computational work. These results suggest that the self-assembly of nanostructured morphologies can play an important role in controlling mechanochemical activation in polymeric materials and provide insights into how polymer composition and morphology impact molecular-scale force distributions.

## Introduction

Polymer mechanochemistry has attracted significant interest in the past decade because it offers an attractive platform for driving chemical transformations by applying mechanical loads to a macroscopic material.^1−4^ In polymer mechanochemistry, a force-responsive molecule, called a mechanophore, is functionalized with polymer chains on opposite sides of the force-responsive bond. These chains act as “handles” that enable transduction of macroscopic forces to the molecular scale, which in turn drives the desired chemical response. A wide range of mechanophores have been developed, enabling polymeric materials to exhibit a useful and diverse set of responses to applied force. Mechanophores that change color in response to force, for example, provide an efficient way of monitoring the molecular-scale forces experienced by a material and have practical applications in damage reporting and stress- or strain-sensing materials.^5−8^ Mechanophores with other chemical responses to force similarly have uses in self-healing materials, synthetic chemistry, and drug delivery, to name only a few applications.^2,9−12^ A significant challenge in the development of efficient mechanochemical materials, however, is that only a limited fraction of the mechanophores are typically activated when the material is deformed.^13−15^ Thus, it is critical to understand, from a fundamental perspective, what features of polymer networks determine the mechanochemical responses of the materials and how network structure can be tuned to optimize this response.

To date, many experiments in polymer mechanochemistry have focused on the architectural features, such as molecular weight and branching, that affect mechanochemical activation in solution.^3,16−18^ However, much less is understood about the structural features necessary for driving activation in bulk polymers, particularly in cross-linked networks and self-assembled network-like polymer structures. In un-cross-linked linear polymers, achieving efficient activation requires careful tuning of the glass transition temperature of the material; when the material is too far below its glass transition temperature, it fractures before substantial activation occurs, while when it is too far above its glass transition temperature, the chains relax before they can transmit enough stress to the mechanophore to activate it.^19^ The activation efficiency can be increased using more complex polymer architectures, such as star, comb, or bottlebrush polymers.^18,20−22^ Linking polymer chains into chemically or physically cross-linked networks offers an alternative route to facilitate efficient stress transmission between the chains.^23−25^ A number of groups have shown that mechanochemical activation can be driven in networks with mechanophores in the cross-linkers.^26−28^ However, modeling based on rubber elasticity theory and coarse-grained simulations of random networks both suggest that inhomogeneity in the cross-link distribution leads to stress concentration in a small number of chains, which in turn decreases the overall activation efficiency.^29,30^

Self-assembled triblock copolymers are an attractive platform for investigating how network structure affects mechanochemical activation because they can be designed such that the elastically effective strands in the midblock are the same length throughout the material, and the network connectivity can be tuned by changing the block fractions, which in turn change the self-assembled morphology.^31,32^ A small number of reports have offered preliminary evidence that block copolymer self-assembly can indeed be used to facilitate mechanochemical activation in polymeric materials.^33,34^ For example, Jiang et al. incorporated a mechanochemically active spiropyran unit into the center of polystyrene-*block*-poly(*n*-butyl acrylate)-*block*-polystyrene (PS-*b*-PnBA-SP-PnBA-*b*-PS) triblock copolymers and reported that mechanochemical activation was observed in the polymer samples that microphase segregated.^33^ However, only a small fraction of the polymers investigated in this work formed microphase-segregated structures, likely due to the relatively small Flory–Huggins interaction parameter for the PS-PnBA system (χ = 0.007)^35^ and the low molecular weights of the polymers, making it difficult to draw systematic conclusions about morphology–activation and composition–activation relationships. Similarly, Ramirez et al. functionalized a polystyrene-*block*-poly(1,4-butadiene)-*block*-polystyrene triblock copolymer with *gem*-dibromocyclopropane mechanophores in the butadiene midblock and observed that the mechanochemical activation was enhanced in the functionalized triblock relative to mechanophore-functionalized polybutadiene alone.^34^ However, only one triblock copolymer composition was investigated in this work, limiting systematic analysis of the role of composition and morphology. Thus, while block copolymers show significant promise in promoting mechanochemical activation, it is critical to understand how the composition and morphology of the block copolymers affect the efficiency of the activation process in order to provide useful guidance in designing functional polymeric materials.

Recently, we used coarse-grained molecular dynamics simulations to systematically investigate how polymer composition and morphology affect mechanochemical activation in triblock copolymers.^36^ In these simulations, the polymer was represented by a Kremer–Grest bead–spring model and the mechanophore by a double-well potential in the center of the rubbery midblock. The simulation data revealed that most activation occurs in the tie chains connecting different neighboring glassy domains, with minimal activation occurring in loop chains. As a result, spherical morphologies, which have a higher rubbery fraction and a higher fraction of tie chains, exhibit higher activation at the same true stress. Lamellar morphologies, however, reach higher stresses in comparison to the spherical morphologies and exhibit higher activation at the same strain but exhibit lower activation at the same stress. These predictions suggest that tuning block copolymer composition and morphology should provide a versatile route to tuning mechanochemical activation responses in self-assembled materials.

Here, we test these predictions in a series of poly(methyl methacrylate)-*b*-poly(*n*-butyl acrylate)-*b*-poly(methyl methacrylate) triblock copolymers containing a force-responsive spiropyran unit in the center of each polymer chain (PMMA-*b*-PnBA-SP-PnBA-*b*-PMMA). This polymer system was chosen because it has a high enough χ value (χ = 0.047)^35^ to ensure phase separation at all compositions investigated in this work, and tuning the PMMA block length while keeping the PnBA block constant yielded samples with spherical and cylindrical microphase-segregated morphologies. We investigated the mechanochemical activation behavior of these polymers using simultaneous tensile tests and optical measurements and found that, consistent with our previous simulations, higher activation is obtained in samples with higher glassy (PMMA) block content when compared at the same strain, but with lower PMMA content when compared at the same stress. Interestingly, while changes in the self-assembled morphology do appear to influence the rate of activation at low strain, the experiments suggest that the overall activation is dominated primarily by the glassy block content of the materials rather than by their specific morphologies. These results provide experimental evidence for the composition-dependent activation predicted in our previously published simulations and bring new insights into the additional features of real experimental systems that influence activation. Together, these results highlight the role of microphase segregation and nanoscale ordering in determining the molecular-scale force responses of polymeric materials and suggest that controlling microphase segregation is a promising avenue for tailoring the mechanochemical responses of materials to obtain precisely tuned responses to stress and strain.

## Experimental Methods

### Materials

2-Bromopropionic acid, acetone, acetonitrile, anhydrous ethyl ether, hexanes, hydrochloric acid (37%), anhydrous magnesium sulfate, methanol, methylene chloride (DCM), potassium hydroxide, sodium bicarbonate, sodium chloride, sodium hydroxide, HPLC-grade tetrahydrofuran (THF), toluene, and *N*,*N*′-dicyclohexylcarbodiimide (≥99%) (DCC) were purchased from Fisher Scientific. Ethanol was purchased from Decon Laboratories. 1,4-Dioxane, 2-bromoethanol (95%), 4-(dimethylamino)pyridine (≥99%) (DMAP), benzyl alcohol, *n*-butyl acrylate (nBA), copper(I) bromide (99.999%), copper(I) chloride (>99.995%), ethyl acetate (99.5%), methyl methacrylate (MMA), and *N*,*N*,*N*′,*N*″,*N*″-pentamethyldiethylenetriamine (PMDETA) were purchased from Sigma-Aldrich. 2,3,3-Trimethylindolenine (>97.0+%) and 3-chloromethyl-5-nitrosalicylaldehyde were purchased from TCI Chemicals. Phosphotungstic acid was purchased from Electron Microscopy Sciences. Milli-Q water (18.2 MΩ·cm) was obtained from a Synergy water purification system (MilliporeSigma). The monomers (nBA and MMA) were filtered through activated neutral alumina immediately before use to remove inhibitor. The initiator, SPBr_2_, was synthesized following the literature procedure (see the Supporting Information). All other reagents were used as received unless otherwise noted.

### Synthesis of PMMA-*b*-PnBA-SP-PnBA-*b*-PMMA (MBM) by ATRP

**Scheme 1:**
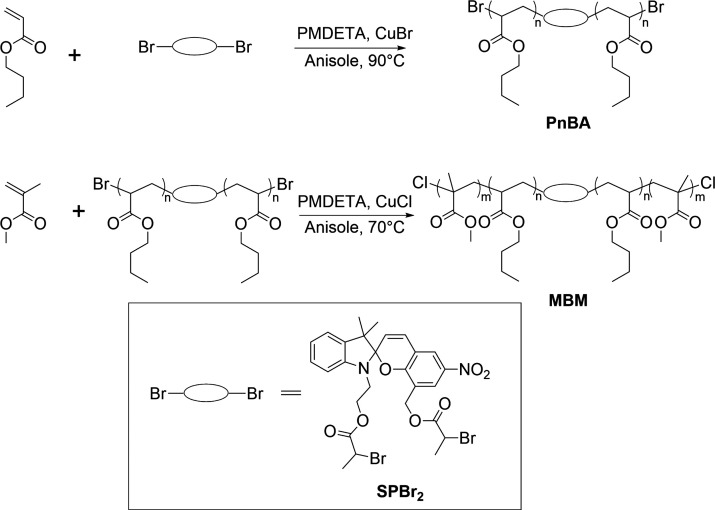
Synthesis of Spiropyran-Containing MBM Triblock Copolymers

MBM was synthesized via a two-step atom-transfer radical polymerization (ATRP) process, as summarized in Scheme 1.^37^

Briefly, a poly(*n*-butyl acrylate) (PnBA) macroinitiator was first synthesized from initiator SPBr_2_, yielding PnBA with a force-responsive spiropyran unit in the center of each chain. This macroinitiator was then chain-extended with methyl methacrylate to yield PMMA-*b*-PnBA-SP-PnBA-*b*-PMMA (MBM) triblock copolymers with a spiropyran unit in the center of the PnBA midblock. Detailed synthetic protocols for each of these steps are provided below.

### Synthesis of Poly(*n*-butyl acrylate) Macroinitiator (PnBA-SP-PnBA)

The PnBA macroinitiator was synthesized from the difunctional spiropyran initiator SPBr_2_ via ATRP. Copper(I) bromide (175 mg, 1.17 mmol) was first added to a Schlenk flask equipped with a magnetic stir bar. This flask was sealed with a septum and immersed in liquid nitrogen. nBA (54.5 g, 426 mmol), SPBr_2_ (346 mg, 0.531 mmol), and PMDETA (222 μL, 1.06 mmol) were then dissolved in 15.2 mL of anisole and transferred to the flask via syringe. The reaction mixture was frozen and degassed via three freeze–pump–thaw cycles. After the last pump cycle, the flask was filled with argon and placed in an oil bath at 90 °C. The reaction was then stirred for 3.5 h, and aliquots were withdrawn periodically to monitor the reaction progress and monomer conversion by ^1^H NMR. After the reaction reached ∼70% conversion, the reaction mixture was exposed to air and cooled in an ice bath to quench the polymerization. The polymer was then precipitated into a 50% ethanol–water mixture over an ice bath. The precipitated polymer was dissolved in minimal methylene chloride and passed through a silica column to remove the remaining copper catalyst. The polymer was then precipitated a second time into a 50% ethanol–water mixture, dissolved in methylene chloride, and passed through a column packed with silica gel to remove any remaining water. Finally, the product was concentrated under reduced pressure, yielding an orange-brown viscous liquid, and dried under vacuum at 50 °C until a constant weight was reached. The resulting PnBA macroinitiator was characterized by ^1^H NMR and size exclusion chromatography (SEC) in THF at 40 °C on an instrument (TOSOH EcoSEC HLC-8320GPC) equipped with multiangle light scattering (Wyatt Technology DAWN8+) and refractive index (TOSOH) detectors. The molecular weight of the polymer was determined using a d*n*/d*c* value of 0.067.^38^

### Synthesis of PMMA-*b*-PnBA-SP-PnBA-*b*-PMMA (MBM)

MBM triblock copolymers were synthesized by chain extension of the PnBA macroinitiator described above. Copper(I) chloride (43.4 mg, 0.434 mmol) was placed in a Schlenk flask, and the sealed flask was immersed in liquid nitrogen. MMA (43.4 g, 434 mmol), PnBA macroinitiator (18.9 g, 0.270 mmol, *M*_n_ = 70 kg/mol), and PMDETA (88.2 μL, 0.423 mmol) were then dissolved in anisole (62 mL) and transferred to the Schlenk flask via syringe. The flask was degassed via three freeze–pump–thaw cycles, and the mixture was placed in an oil bath at 70 °C to initiate polymerization. The reaction conversion was monitored by NMR over a period of 9 h. When the conversion needed for each target PMMA molecular weight was reached, a large aliquot was withdrawn and quenched by exposing the aliquot to air and cooling it to room temperature. Each aliquot was then precipitated into ethanol in an ice bath, redissolved in methylene chloride, passed through a silica column, and precipitated into ethanol once again. The polymer was collected and dried under vacuum to yield the target MBM triblock copolymer as a light-brown rubbery solid. The polymers were characterized by ^1^H NMR and size exclusion chromatography (SEC) as described above. The d*n*/d*c* value for the MBM triblock copolymer was calculated from the d*n*/d*c* values of PnBA and PMMA (0.067 and 0.085, respectively),^38^ and the weight fractions of the PMMA and PnBA blocks were obtained from ^1^H NMR (see the Supporting Information).

### Tensile Sample Preparation

To prepare self-assembled samples of the MBM triblock copolymers for tensile testing, a concentrated polymer solution (8 mL, ca. 17 wt % in toluene) was deposited in a PTFE mold and allowed to dry by slow evaporation at room temperature for 1 day, followed by annealing under dioxane vapor at 50 °C for another 2 days.^39,40^ The samples were then dried under vacuum overnight until they reached a constant weight, yielding films with thicknesses of 0.50 ± 0.10 mm. Tensile samples were then prepared by die-cutting the polymer film with a custom micro tensile bar dog-bone mold with a gauge length of 15 mm (see the Supporting Information).

### TEM Sample Preparation

The undeformed portions of the tensile samples were cut into ultrathin sections (ca. 70–90 nm thick) with a diamond knife on a Leica UC6/FC6 ultramicrotome under cryogenic conditions at −120 °C. Thin sections were collected on 400 mesh Carbon B copper grids (0814-F, Ted Pella), and the PMMA regions were then stained by floating the samples on an aqueous solution containing 2 wt % phosphotungstic acid (PTA) and 2 wt % benzyl alcohol at room temperature for 5 min.^35^ Stained samples were imaged using a Thermofisher FEI Talos F200C electron microscope operated at 200 kV at various magnifications to determine the morphology of the triblock copolymers.

### Tensile Tests and Optical Measurements

Tensile tests were performed on a custom-built tensile tester consisting of two translation stages (Aerotech PRO165SL) that translate simultaneously in opposite directions and a 50 lb capacity load cell (Honeywell Model 31). A diagram of this tensile tester, which was inspired by Celestine et al.,^41^ is provided in the Supporting Information. For absorption measurements, the samples were illuminated from behind using a white LED pad (A4-DWT, Tikteck) and imaged using a monochromatic camera (DCC1240M, Thorlabs) equipped with a bandpass filter with a transmission range of 550–630 nm (89-811, Edmund Optics).

The tensile test protocol was adapted from the American Society for Testing and Materials standard (ASTM D638).^42^ Prior to tensile tests, the tensile samples were irradiated using a green flashlight (530 nm, JOYLIT) at room temperature for 2 h to drive any merocyanine back to its SP closed-ring form.^43^ The samples were then loaded onto the tensile tester between the grips. For samples with low PMMA content, the tensile sample was first cooled over dry ice for 15 min to facilitate handling, loaded onto the tensile tester while it was still cold, and then warmed to room temperature before testing. The stages were then each translated at a velocity of 0.085 mm/s while recording video. The experiment stopped when the sample broke or fractured into two parts. During the tensile tests, the stress–strain curves and videos were recorded using a custom-built LabVIEW interface. The video frames were later analyzed using Python to extract the activation profile of the triblock copolymer under deformation, as described in the Results section.

## Results

### Polymer Synthesis

The spiropyran-containing PnBA macroinitiator was first synthesized following the procedure described above. SEC indicated that this PnBA macroinitiator had a number-average molecular weight of 70 kg/mol and a dispersity of 1.14, indicating good control during ATRP from the SPBr_2_ initiator. Five MBM triblock copolymers were then synthesized from this PnBA macroinitiator. The compositions of the resulting polymers are summarized in Table 1. The polymers are labeled as M_*x*_B_*y*_M_*x*_, where *x* indicates the number-average molecular weight of each PMMA end-block and *y* indicates the number-average molecular weight of the PnBA midblock. As seen in this table, all MBM triblock copolymers had the same 70 kg/mol PnBA midblock, and PMMA end-blocks ranging from 13 to 45 kg/mol, yielding volume fractions of PMMA (*f*_PMMA_) from 0.21 to 0.50 and χ*N* values ranging from approximately 40 to 70, respectively. These values were targeted to obtain different microphase-segregated morphologies across the block copolymer phase diagram. For all polymers, the molecular weights estimated from NMR match those calculated by SEC to within ∼10%. The dispersities of the MBM triblock copolymers increased slightly with PMMA block length (from 1.21 for M_13_B_70_M_13_ to 1.29 for M_35_B_70_M_35_) but generally remained low enough to indicate reasonable control of the polymerization. However, for the highest molecular weight sample (M_45_B_70_M_45_), a lower molecular weight shoulder and an increase in dispersity were observed, suggesting some loss in control due to the increase in viscosity of the reaction mixture.

**Table 1:** Compositions of MBM Triblock Copolymers Synthesized in This Work

polymer	*M*_n,NMR_ (kg/mol)	*M*_n,SEC_ (kg/mol)	*Đ*	*f*_PMMA_	χ*N*^a^	morphology
B_70_^b^	72	70	1.14			
M_13_B_70_M_13_	91	97	1.21	0.21	38	spherical
M_20_B_70_M_20_	101	110	1.21	0.29	45	spherical
M_25_B_70_M_25_	107	121	1.22	0.33	49	cylindrical
M_35_B_70_M_35_	129	140	1.29	0.44	59	cylindrical
M_45_B_70_M_45_	145	159	1.39	0.50	68	cylindrical

a χ = 0.047 at room temperature.^35^

b The subscripts in each abbreviation correspond to the number-average molecular weight of each block in kg/mol, as measured by SEC.

### Morphology

**Figure 1:**
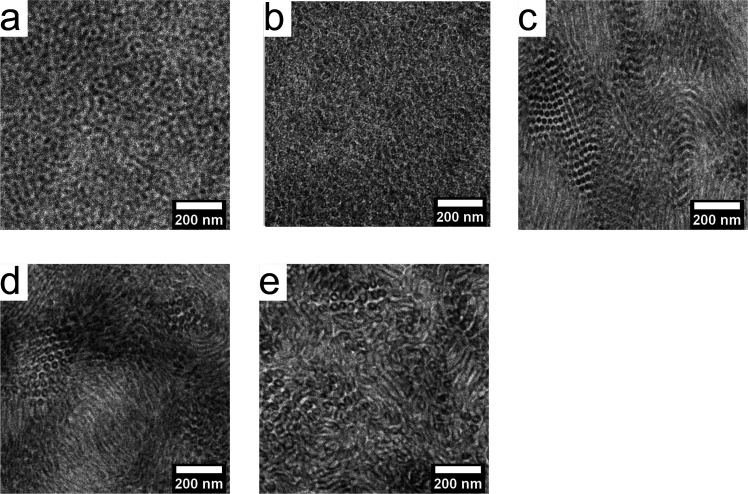
TEM images of MBM triblock copolymers stained with benzyl alcohol and phosphotungstic acid. PMMA microdomains are shown in black, and PnBA is shown in white: (a) M_13_B_70_M_13_, (b) M_20_B_70_M_20_, (c) M_25_B_70_M_25_, (d) M_35_B_70_M_35_, and (e) M_45_B_70_M_45_.

The block copolymers were solvent-cast and thermo-solvent annealed, as described in the Experimental Methods section, and their morphologies were characterized by transmission electron microscopy (TEM). Representative TEM images for each MBM polymer are shown in Figure 1, and their assigned morphologies are listed in Table 1. In these images, the PMMA microdomains were selectively stained with phosphotungstic acid solution and appear black, while the PnBA domains were unstained and remain white. The images of the M_13_B_70_M_13_ and M_20_B_70_M_20_ samples (Figures 1a,b) show predominantly circular PMMA regions, indicating a spherical morphology, although long-range hexagonal order was not observed. The images of the M_25_B_70_M_25_ and M_35_B_70_M_35_ (Figures 1c,d) show both hexagonally packed circular regions and vertical or horizontal stripes, indicating predominantly cylindrical morphologies. Similar features were observed for M_45_B_70_M_45_ (Figure 1e), but with much less well-defined ordering, possibly due to this polymer’s higher dispersity and/or limitations of the thermo-solvent annealing process for high molecular weight polymers.

### Mechanical Properties

**Figure 2:**
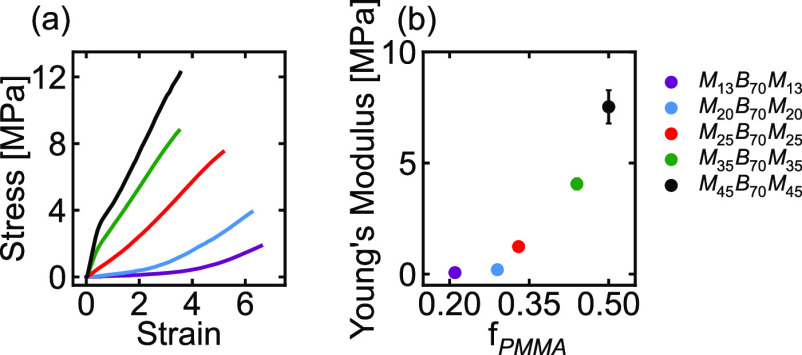
(a) Stress–strain behavior and (b) Young’s moduli of MBM triblock copolymers with different PMMA fractions. Vertical error bars represent the standard deviation of 3 or 4 repeat measurements (see the Supporting Information). For the first four samples, the error bar is smaller than the symbol size. Numeric values of the moduli for all polymers are reported in the Supporting Information. All stress–strain values are engineering stress and engineering strain unless otherwise noted.

Tensile tests were then performed on all five MBM triblock copolymer samples. The resulting stress–strain curves are shown in Figure 2a. As seen in this figure, the polymers with the lowest volume fraction of PMMA (M_13_B_70_M_13_ and M_20_B_70_M_20_) behaved as elastomeric rubbers and exhibited slight strain stiffening around strains of 3 and 4 before fracturing around a strain of 7. The samples with higher PMMA content (M_35_B_70_M_35_ and M_45_B_70_M_45_) were less extensible and exhibited only a short linear regime before yielding at strains of ∼0.5. The moduli of the MBM triblock copolymers were obtained from a linear fit of the low-strain portion of each stress–strain curve. As seen in Figure 2b, the Young’s moduli of the samples increased with increasing volume fraction of PMMA, as expected. Stress–strain curves and moduli were reproducible across multiple tensile samples for each polymer (see the Supporting Information). We note that the B_70_ midblock alone was too soft to characterize as a “no morphology” control but, as a homopolymer melt far above its glass transition temperature, is expected to undergo negligible activation^19^ and so was omitted from the tensile and activation analyses.

### Activation Behavior

**Figure 3:**
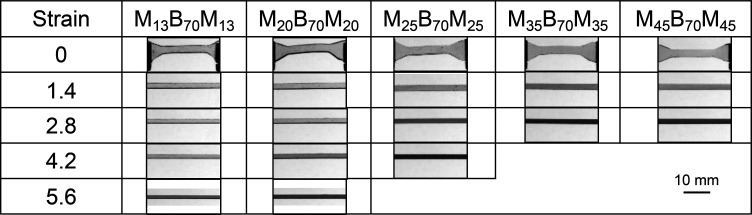
Images of dog-bone samples taken at different strain values.

The activation of the mechanophores in each sample was monitored by recording a video of the samples during tensile deformation. Representative frames from videos of each sample are shown in Figure 3. As seen in these images, the samples with higher PMMA block fractions changed color, indicating activation of the spiropyran mechanophores, at significantly lower strains than those with lower PMMA block fractions. To quantify the relative activation of each sample, a region of interest was selected from within the center of the dog-bone image. The average intensity in this region was calculated for each frame of the video. This intensity was then divided by the background intensity and converted to the corresponding absorbance value. The absorbance at each time point was divided by the instantaneous sample thickness, background-subtracted to account for the small absorbance of the unstrained sample at the beginning of the run, and divided by the molar extinction coefficient of the activated mechanophores (see the Supporting Information) to yield the concentration of activated mechanophores at each time point during the tensile experiment. This concentration was finally divided by the total concentration of mechanophores in the sample (calculated from the density of the polymer divided by its number-average molecular weight) to yield the percent activation. We note that the reported activation percentages depend on the extinction coefficient, which was measured for UV-activated (rather than strain-activated) samples (see the Supporting Information). Because the same molar extinction coefficient was used to analyze all samples, however, any deviations from the actual activation percentages are consistent from sample to sample and do not affect any of the trends reported in this work.

**Figure 4:**
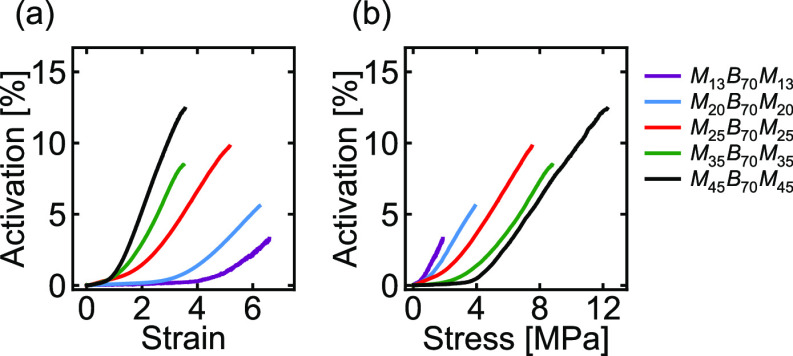
(a) Activation–strain behavior and (b) activation–stress behavior of MBM triblock copolymers with different PMMA fractions.

The resulting activation curves are shown in Figure 4. When the activation is plotted as a function of strain, as shown in Figure 4a, the activation appears to occur earlier in samples with higher PMMA fractions. On the other hand, when the activation is plotted as a function of stress, as shown in Figure 4b, the activation appears to occur earlier in samples with lower PMMA fractions. Samples with higher PMMA fractions generally reached a higher overall activation before fracturing than the samples with lower PMMA fractions. Only minimal reversion of the spiropyran was observed when samples were held under constant strain over the duration of the tensile experiments, indicating that deactivation was suppressed under tensile force (see the Supporting Information).

**Figure 5:**
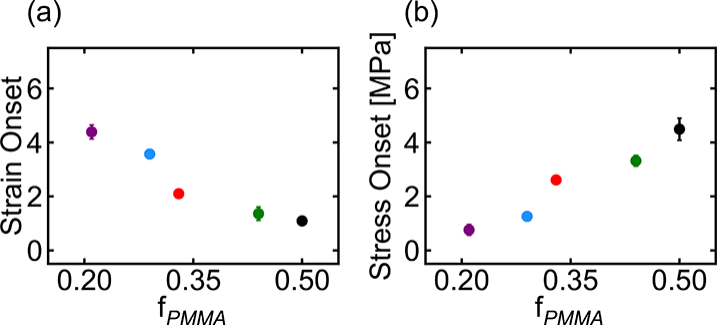
(a) Strain onset point and (b) stress onset point for MBM triblock copolymers with different PMMA fractions. Error bars represent the standard deviation of 3 or 4 repeat measurements (see the Supporting Information). For points that appear to be missing error bars, the error bar is smaller than the symbol size. Numeric values of the onset points for all polymers are reported in the Supporting Information.

**Figure 6:**
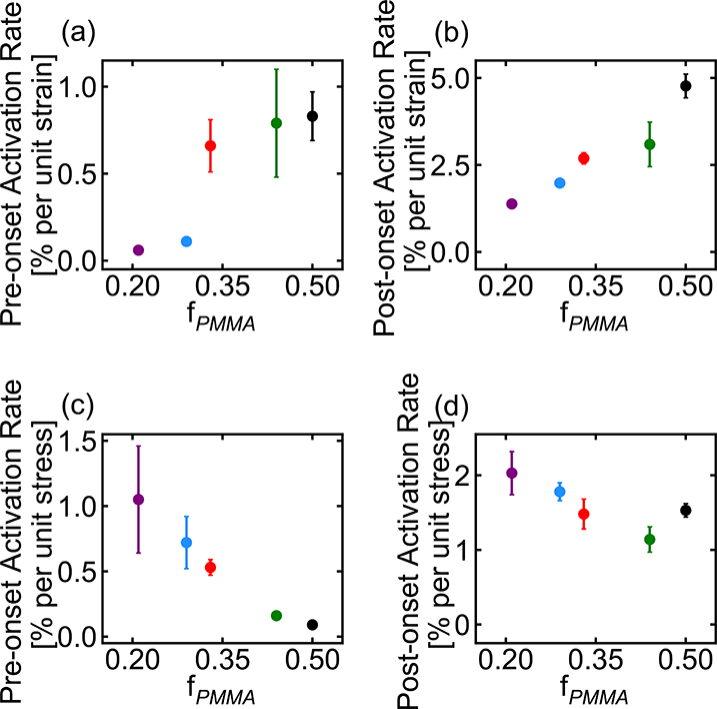
Activation rates before (a, c) and after (b, d) the onset point (a, b) per unit strain and (c, d) per unit stress for MBM triblock copolymers with different PMMA fractions. Error bars represent the standard deviation of 3 or 4 repeat measurements (see the Supporting Information). For points that appear to be missing error bars, the error bar is smaller than the symbol size. Numeric values of the activation rates for all polymers are reported in the Supporting Information.

In both the activation–strain and activation–stress curves, a distinct onset of activation is observed for each polymer composition. The onset point was determined to be the point at which lines fitted to the pre-onset (low activation) and post-onset (high activation) regimes intersected (see the Supporting Information). The onset points, as well as the slopes of the activation curves before and after the onset point (which reflect the activation “rates” per unit strain or per unit stress), are shown in Figures 5 and 6. As seen in these data, the onset point moves to lower strain but higher stress as the fraction of PMMA in the samples increases. The initial (pre-onset) activation rate increases with *f*_PMMA_ in the activation–strain curves but decreases with *f*_PMMA_ in the activation–stress data, while the post-onset activation rate increases with PMMA fraction in the activation–strain data but varies little in the activation–stress plots. Interestingly, the initial (pre-onset) activation rate also exhibits an apparent discontinuity between PMMA fractions of 0.29 and 0.33 in the activation–strain curves, corresponding to the transition from spherical to cylindrical morphologies; slight deviations from linearity are also observed in the onset points in this region, but not in the other pre- or post-onset activation rate plots.

## Discussion

Understanding how network structure and topology affect the transduction of macroscopic forces to the molecular scale is critical for designing efficient polymeric materials for mechanochemical activation. Here, we use block copolymers as a platform for investigating structure–activation relationships in polymeric materials. As described above, we synthesized a series of MBM triblock copolymers containing identical (spiropyran-containing) PnBA midblocks connected to PMMA end-blocks of varying lengths. These polymers self-assembled into well-defined nanostructured morphologies, enabling us to systematically investigate how the compositions and morphologies of triblock copolymers and, more broadly, different network structures affect mechanochemical activation in polymers under tensile deformation.

The MBM system was chosen for this work because it has a relatively high interaction parameter (χ = 0.047),^35^ which drives microphase segregation of the polymers into elastomeric materials with rubbery PnBA chains connecting rigid, glassy PMMA domains.^44^ For the degrees of polymerization investigated in this work, χ*N* ranged from 40 to 70. Critically, all of these χ*N* values are well above the order–disorder transition for triblock copolymer melts,^45^ and all samples were observed to assemble into microphase-segregated nanostructures following a mild thermo-solvent annealing process. Interestingly, however, the obtained nanostructures differed somewhat from those predicted by self-consistent field theory (SCFT).^46^ According to the phase diagram predicted by SCFT, the morphology of M_13_B_70_M_13_ (*f*_PMMA_ = 0.21) was expected to be spherical, M_20_B_70_M_20_ and M_25_B_70_M_25_ (*f*_PMMA_ = 0.29 and *f*_PMMA_ = 0.33) to be cylindrical, and M_35_B_70_M_35_ and M_45_B_70_M_45_ (*f*_PMMA_ = 0.44 and *f*_PMMA_ = 0.50) to be lamellar. However, TEM images revealed that the samples formed only spherical and cylindrical morphologies, even up to PMMA fractions of 0.50, and did not exhibit substantial long-range order, suggesting that kinetic trapping of the morphology during thermo-solvent annealing may prevent the polymers from reaching their equilibrium morphologies.^47^ Although thermal annealing, in which the samples are annealed above their order–disorder transition temperature, is generally the most robust method for obtaining equilibrated phase-segregated morphologies in block copolymers,^48^ we observed a significant broadening of the molecular weight distribution of spiropyran-containing triblocks in preliminary experiments when they were thermally annealed at temperatures above 180 °C, likely due to thermal degradation of the spiropyran unit. While we suspect that kinetic trapping during thermo-solvent annealing led to the apparent shift in the phase behavior observed here, we note that similar trends were observed in at least one prior report on the self-assembly of MBM triblock copolymers, where the deviation from SCFT was attributed to the relatively high dispersity of the PMMA end-blocks.^35^ While the dispersities of the polymers investigated in our work are significantly lower than those reported by Ruzette et al., our MBM triblocks are still not perfectly monodisperse, with somewhat asymmetric molecular weight distributions, and similar dispersity effects may also play a role.^35,49^

As seen in the activation data presented in Figures 3 and 4, all five polymers did indeed exhibit a mechanochemical response (observed as a color change) in response to applied force, indicating that the glassy domains do serve as effective physical cross-links and facilitate transduction of macroscopic stress to drive chain stretching and mechanochemical activation at the molecular scale. Several clear trends were observed in the activation data as a function of PMMA block fraction: First, as the PMMA fraction of the polymers increased, the total activation achieved before fracture generally increased. Second, as the PMMA fraction increased, the strain at which the activation turned on (the “onset”) increased, while the stress at which the activation turned on decreased. Interestingly, both the pre-onset activation rate and the strain and stress onset points show evidence of a slight discontinuity between *f*_PMMA_ = 0.29 and *f*_PMMA_ = 0.33, corresponding to the transition from spherical to cylindrical morphologies; while this suggests that morphology does play a role in activation, the activation appears to be dominated primarily by the changes in *f*_PMMA_ and the overall modulus of the material. We note that, in our samples, increasing *f*_PMMA_ does also increase the total molecular weight of the polymers and that molecular weight increases have been correlated to increased activation in homopolymer systems.^16,18^ Here, however, the relevant molecular weight is the molecular weight of the elastically effective PnBA strands, which was constant in all five block copolymers. PnBA is also, at room temperature, a homopolymer melt far above its glass transition temperature and as such is expected to undergo negligible activation regardless of chain length.^19^ The changes in activation with changes in *f*_PMMA_ are thus associated primarily with changes in the self-assembled morphology and modulus of the material, rather than with changes in the molecular weight of the polymer.

When comparing the experimental results to our previous simulation work, we find that similar trends in both mechanical properties and mechanochemical activation are observed across both approaches. In the experimental data, we observed that as the glassy fraction (*f*_PMMA_) increased, the materials became stiffer (Figure 2); this result is in agreement with our previous simulations, which showed that increasing *f*_PMMA_ generally increased the stress required to reach each strain.^36^ In the experimental data, we also found that increasing *f*_PMMA_ led to an increase in overall activation and an increase in the activation observed at a given strain, but a decrease in the activation observed at a given stress (Figure 4). These results are consistent with our findings from the previous MD simulations that as the glassy component of the triblock copolymer increases, the samples bear higher stresses under the same extent of deformation, which results in higher activation.^36^ Additionally, the observed decrease in activation efficiency at a given stress with increasing PMMA fraction is likely due to the fact that polymer chains are less likely to bridge between different glassy domains as *f*_PMMA_ increases,^46^ which is consistent with the prediction from our computational work that activation primarily occurs in the tie chains in these materials.^36^ Taken together, the general agreement in the mechanical properties and mechanochemical activation behaviors observed between this experimental system and our previous computational model validates the predictions from the MD simulations and provides further insights into how macroscopic force affects mechanochemical activation on the molecular scale.

Despite good general agreement between the experiments and simulations, however, we note that there are several differences between the experimental and simulation results, which may indicate a need for further development of the simulation model. First, with respect to the mechanical properties, the range of strains over which the stress–strain curves are linear is larger in the experiments than in the simulations, and this range narrows with increasing *f*_PMMA_, which was not observed in the simulations. Additionally, the magnitude of the modulus increase with increasing *f*_PMMA_ was much smaller in the simulations than in the present experiments. These discrepancies may result from the unrealistically high strain rates and the short time scales used to access large deformations in the MD simulations.^50^ The glassy regions are also much more ductile in the simulation model than in the experimental system, which may make it easier for the glassy regions to relax.^51^ Second, with respect to the activation behavior, while replotting the data from Figures 2a and 4 in terms of true stress and true strain did not substantially change the trends observed in the onset points or activation rates, all of the experimental samples collapsed onto essentially one activation–true stress curve (see the Supporting Information). The simulations, on the other hand, predicted a substantial shift in this curve with increasing *f*_PMMA_. This mismatch may be because the experiments only accessed spherical and cylindrical morphologies, while the biggest difference in the simulations was observed as the samples transitioned from cylindrical to lamellar. However, differences in the force distributions resulting from the randomized domain orientations in the experimental polymer samples, in contrast to the well-aligned samples generated in the MD simulations, as well as domain reorientations during deformation in the experimental system, may also play a role. Finally, we note that the simulations were run on perfectly monodisperse samples, while the experimental samples have low, but non-negligible, dispersities. We suspect that the short and long chains in polydisperse samples may activate at different times, complicating interpretation of mechanochemical activation in these materials, but further work will be needed to test this hypothesis. Thus, while the overall trends predicted by the simulations are consistent with those observed in the present experiments, these differences do suggest that the simulations do not yet capture the full complexity of the experimental systems, and further work on developing the simulation models will be necessary to fully understand all of the observed experimental trends.

Taken together, our results show that block copolymers are an attractive platform for driving mechanochemical activation in polymeric materials. Changing the composition and morphology of the polymer changes both the mechanical properties and the activation–strain and activation–stress profiles, which should allow the activation behavior to be tuned to meet the needs of different applications. Additionally, our work shows that the overall trends in activation behavior are well-predicted by coarse-grained molecular dynamics simulations. The simulations and experiments provide complementary information about the force responses in these systems: simulations provide insight into the molecular-scale mechanisms underlying the experimentally observed trends in activation, while the experiments provide critical information about the features of real polymer systems that are not well-described by current simulation models but play a important role in activation. Triblock copolymers are chemically and physically complex, making them an ideal test system for investigating force transduction both experimentally and computationally, and further feedback between experiments and simulations will advance understanding of both macroscopic force responses and mechanochemical activation in these materials.

There may be a number of interesting avenues for investigating these complexities in future work. First, while the experimental data suggest that the picture from simulations in which most activation occurs in the tie chains is correct, the present experiments do not allow this prediction to be tested directly. Experiments in which the solvent quality is varied during the solvent casting and thermo-solvent annealing step of the sample preparation to change the tie chain fraction in the materials may provide insight into how the activation profiles depend on the tie chain population. Second, while simulations suggest that domain orientation strongly influences activation profiles and that much of the activation happens at areas of high local deformation, the present experiments cannot probe the roles that domain orientation and reorientation, and rearrangement of grain boundaries, play in the activation process. Future work on prealigned samples or experiments employing simultaneous tensile, optical, and scattering measurements to probe domain alignment may provide insight into this question. Third, the experimental systems are inherently more complex than those modeled in our prior work because the synthesized block copolymers are not perfectly monodisperse. Investigation of the role of the molecular weight distributions of the polymers will be critical for understanding how the presence of chains of different lengths in the same sample influences the activation process. Fourth, while the present data suggest that both morphology and overall glassy content play a role in the observed mechanochemical activation, further experiments on samples with the same morphology but different moduli and/or same moduli but different morphologies will be critical for determining the precise role of both morphology and glassy content in driving activation in these materials. And finally, while the present experiments focus only on polymers with mechanophores in the middle of the rubbery blocks, it may be interesting to investigate how the activation profiles depend on the placement of the mechanophores within the chains. Such experiments would effectively allow investigation of how the local tensions vary at different points along the polymer chains and should provide insights into how chain tensions change near the glassy–rubbery interface. These experiments, and others, should not only advance fundamental physical understanding of how force is transmitted through nanostructured block copolymers, but should also provide both valuable data for enriching and improving simulation models and new insights into how to design functional mechanochemically responsive materials.

## Conclusions

In conclusion, we have shown that polymer composition and morphology can strongly impact the mechanochemical activation behaviors of nanostructured triblock copolymers. Using MBM triblock copolymers with identical spiropyran-containing rubbery PnBA midblocks and glassy PMMA end-blocks of varying lengths, we were able to obtain polymer samples with spherical and cylindrical morphologies and studied their mechanochemical activation behaviors using simultaneous tensile tests and optical measurements. We found that as the fraction of the glassy PMMA blocks increased, the activation turned on at higher stress but at lower strain, with some evidence of a change in activation at the transition from spherical to cylindrical morphologies. These findings generally agree with the predictions from our prior simulation work; however, differences between the experiments and simulations were observed in some of the mechanical properties and activation–stress curves. We attributed these differences to the fast strain rates and ductile glassy blocks used in the simulations and to the randomized domain orientations and dispersities of the experimental samples, and anticipate that further developing the experiments and simulations to address these differences should be a fruitful direction for future work. Overall, this work demonstrates the importance of polymer composition, morphology, and effective network structure in controlling mechanochemical activation in polymeric materials and should help inform the design of functional materials with targeted mechanochemical responses.

## Supporting Information Available

Details of the spiropyran synthesis, supplemental characterization data (^1^H NMR, HRMS, SEC), details of the tensile setup, and additional tensile data and analysis including thickness normalization, onset point determination, and relaxation of mechanophores under strain (PDF)
