# Correction to “Effect of Polymer Composition and Morphology on Mechanochemical Activation in Nanostructured Triblock Copolymers”

**DOI:** 10.1021/acs.macromol.4c00956

**Published:** 2024-05-08

**Authors:** Zijian Huo, Swati Arora, Victoria A. Kong, Brandon J. Myrga, Antonia Statt, Jennifer E. Laaser

In the original article,^1^ the Acknowledgment incorrectly failed to recognize funding from NSF award number DMR-2143864. The corrected Acknowledgment is

- This work was supported by the National Science Foundation (DMR-1846665, Z.H., S.A., V.A.K., B.J.M., and J.E.L., and DMR-2143864, A.S.). The authors also thank Missy L. Hazen at Penn State Microscopy Facility, University Park, PA, for providing services on TEM imaging.
