# Supplementary material for: Effect of Polymer Composition and Morphology on Mechanochemical Activation in Nanostructured Triblock Copolymers
Source: Macromolecules. 2023 Mar 2;56(5):1845–54. doi: 10.1021/acs.macromol.2c02475 (PMC10018773; doi:10.1021/acs.macromol.2c02475)
Supplement: Supplementary file 1 — ma2c02475_si_001.pdf [file ma2c02475_si_001.pdf]

Supporting Information: Effect of Polymer Composition and Morphology on Mechanochemical Activation in Nanostructured Triblock Copolymers

Zijian Huo,[†] Swati Arora,[†] Victoria A. Kong,[†] Brandon J. Myrga,[†] Antonia Statt,[‡] and Jennifer E. Laaser[*,†]

†*Department of Chemistry, University of Pittsburgh, 219 Parkman Ave., Pittsburgh, PA 15260, United States*

‡*Materials Science and Engineering, Grainger College of Engineering, University of Illinois, Urbana-Champaign, IL 61801, United States*

E-mail: j.laaser@pitt.edu

Phone: (650)804-2020

Contents

1 Synthesis of $SPBr_2$ S-2

2 Characterization Data for MBM Triblock Copolymers S-8

2.1 ^{1}H-NMR S-8

2.2 SEC S-9

3 Tensile Measurements S-11

3.1 Experimental Setup S-11
3.2 Determination of the Extinction Coefficient of Activated Spiropyran S-12
3.3 Thickness Normalization S-14
3.4 Repeatability of Measurements S-15
3.5 True Stress and True Strain S-17
3.6 Summary of Activation Behavior S-18
3.7 Onset Point Determination S-19
3.8 Relaxation of Mechanophores during Tensile Measurements S-20

References **S-21**

1 Synthesis of $SPBr_2$

The spiropyran-based initiator $SPBr_2$ (**5**) was synthesized according to the literature procedure, as shown in Scheme S1.,[S1–S4] and characterized by ^{1}H-NMR on a Bruker 400 MHz NMR spectrometer and high-resolution mass spectroscopy using a Thermo Scientific Q Exactive Orbitrap mass spectrometer in positive ion mode (ESI+).

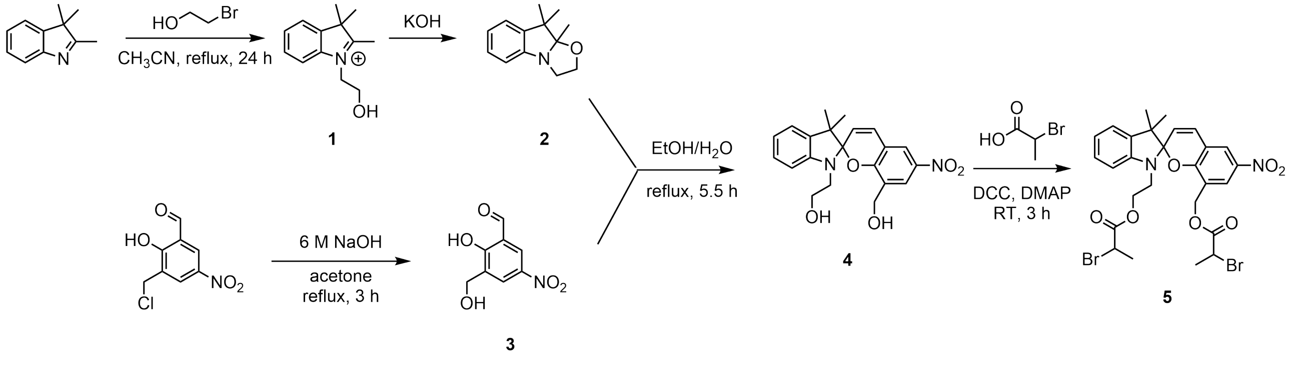

Scheme S1: Synthetic scheme of $SPBr_2$ initiator for ATRP

Synthesis of (1) 2,3,3-trimethylindolenine (4.8 mL, 30 mmol) and 2-bromoethanol (2.7 mL, 38 mmol) were dissolved in acetonitrile (40 mL) and heated to reflux for 18 h. After

refluxing, the reaction mixture was concentrated under reduced pressure to remove acetonitrile. Hexanes (50 mL) were added, and the mixture was stored overnight in the refrigerator to induce crystallization. The resulting mixture was filtered, washed with diethyl ether, and dried under vacuum to yield **1** as a pink solid (4.5 g, 16 mmol, 52 %). ^{1}H-NMR (400 MHz, DMSO-d$_6$, Fig. S1) δ 7.96 (m, 1H), 7.86 (m, 1H), 7.63 (d, 2H), 4.60 (t, 2H), 3.88 (t, 2H), 2.80 (s, 3H), 1.56 (s, 6H).

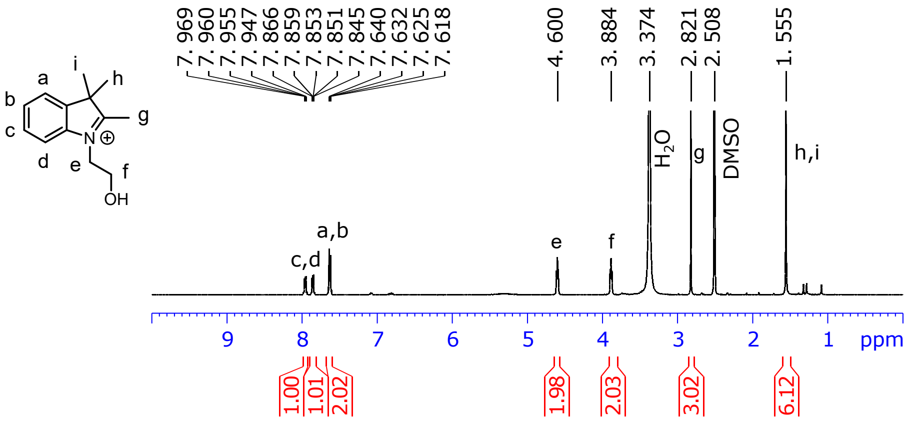

Figure S1: ^{1}H-NMR spectrum of **1** in DMSO-d$_6$ (400 MHz)

Synthesis of (2) Indole **1** (3.0 g, 11 mmol) was converted to **2** by grinding with potassium hydroxide (1.1 g, 20 mmol) in a mortar and pestle for 30 minutes to yield a gold-colored liquid. The crude product was extracted with diethyl ether, and the extracts were washed with saturated sodium chloride solution, dried over anhydrous magnesium sulfate, filtered, and concentrated. The resulting product was then purified by column chromatography (1:3 v/v ethyl acetate:hexanes, R_f = 0.40). The collected fraction was concentrated and dried under vacuum to obtain **2** as a viscous yellow oil (2.0 g, 10 mmol, 90%). ^{1}H-NMR (400 MHz, DMSO-d$_6$, Fig. S2) δ 7.16 (m, 1H), 7.11 (m, 1H), 6.95 (m, 1H), 6.78 (m, 1H), 3.87 (m, 2H), 3.57 (m, 2H), 1.45 (s, 3H), 1.41 (s, 3H), 1.21 (s, 3H).

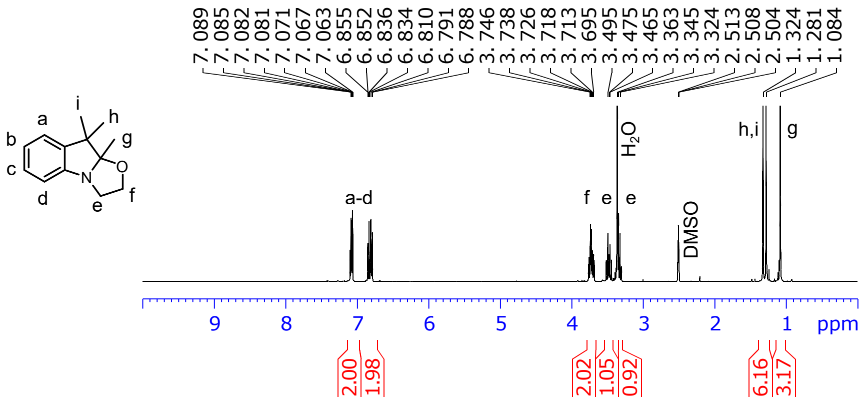

Figure S2: ^{1}H-NMR spectrum of **2** in DMSO-d_6 (400 MHz).

Synthesis of (3) 3-chloromethyl-5-nitrosalicylaldehyde (7.9 g, 37 mmol) was dissolved in acetone (42 mL) and added to a 250 mL round bottom flask with Milli-Q water (12 mL) while stirring. Concentrated sodium hydroxide solution (6.4 mL, 6 M) was added dropwise to the mixture and the system was heated to reflux for 3 h. After refluxing, the reaction mixture was cooled back to room temperature, concentrated to remove acetone, and purified via acid-base precipitation. The concentrated solution was first diluted in 30 mL Milli-Q water, and concentrated sodium hydroxide solution (6 M) was added dropwise until no more precipitate was dissolved. The resulting solution was filtered, and the filtrate was collected. The filtrate was then cooled in an ice bath and acidified using concentrated hydrochloric acid (12 M) until the product precipitated as a light yellow powder. The product was washed with water and collected by vacuum filtration, and was finally dried under vacuum to yield **3** as a white powder (4.5 g, 23 mmol, 62%). ^{1}H-NMR (400 MHz, DMSO-d_6, Fig. S3) δ 10.2 (s, 1H), 8.59 (s, 1H), 8.45 (s, 1H), 4.62 (s, 1H)

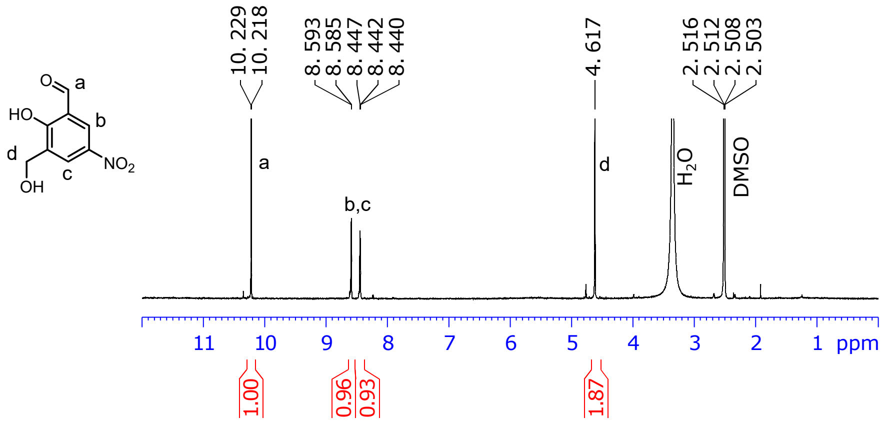

Figure S3: ^{1}H-NMR spectrum of **3** in DMSO-d_6 (400 MHz).

Synthesis of (4) Compound **3** (4.0 g, 21 mmol) was added to Milli-Q water (70 mL) in a 250 mL round bottom flask. Compound **2** (5.2 g, 25 mmol) was then dissolved in ethanol (70 mL) and added to the flask while stirring. The reaction mixture was heated to reflux for 5 h, cooled to room temperature, and concentrated to remove ethanol. The resulting mixture was then separated by column chromatography (1:1 v/v ethyl acetate: hexanes, R_f = 0.40). The collected fractions were concentrated and dried under vacuum to yield **4** as a purple paste (5.5 g, 14 mmol, 68%). ^{1}H-NMR (400 MHz, DMSO-d_6, Fig. S4) δ 8.11 (s, 2H), 7.12 (m, 4H), 6.65 (m, 1H), 6.01 (m, 1H), 4.71 (m, 1H), 4.22 (m, 2H), 3.20 (m, 2H), 1.19 (s, 3H), 1.11 (s, 3H). The product was confirmed by HRMS (ESI) m/z $[M+H]^+$ calculated for $C_{21}H_{22}N_2O_5$ 383.15, found 383.16.

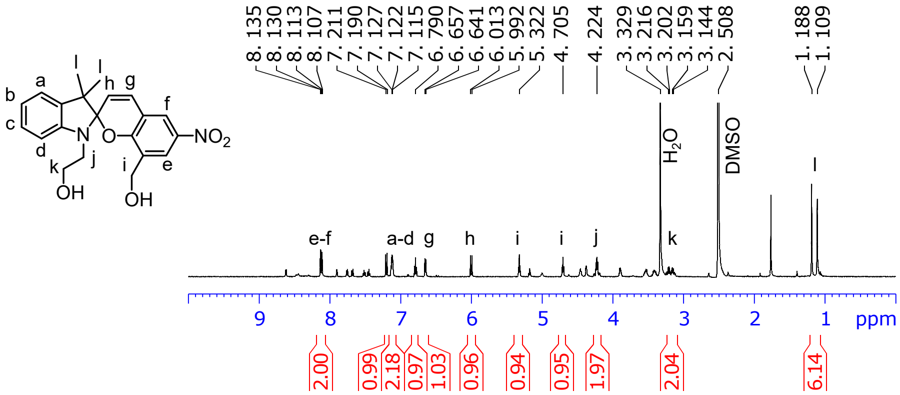

Figure S4: ^{1}H-NMR spectrum of **4** in DMSO-d$_6$ (400 MHz).

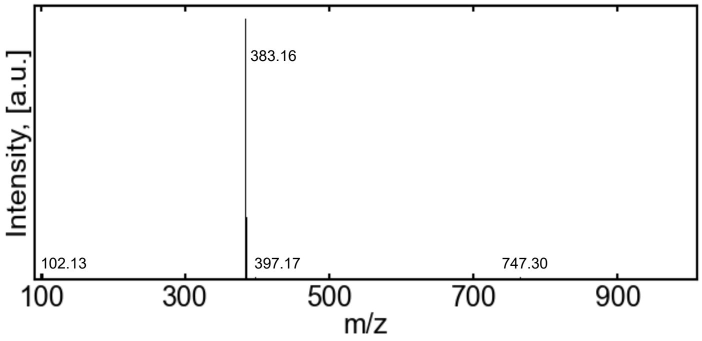

Figure S5: High-resolution mass spectrum of **4**

Synthesis of (5) Dihydroxyspiropyran **4** (0.3 g, 0.78 mmol) was dissolved in methylene chloride (8 mL) in a 25 mL round bottom flask. DCC (0.4 g, 2 mmol) and DMAP (14 mg 0.11 mmol) were dissolved in methylene chloride (1 mL) and added dropwise to the flask while stirring. After stirring for 10 min, 2-bromopropanoic acid (0.4 mL, 4 mmol) was added dropwise to the flask. The resulting mixture was stirred at room temperature for 3 h, after which the precipitate was recovered by vacuum filtration and washed with methylene chloride. The crude product was then concentrated and purified by column chromatography (1:4 v/v ethyl acetate/hexanes, R_f = 0.35). The collected fractions were washed with 1 M sodium bicarbonate solution (50 mL) three times and dried over anhydrous magnesium sulfate to yield initiator **5** as a purple paste (0.15 g, 0.23 mmol, 25%). ^{1}H-NMR (400 MHz,

DMSO-d$_6$, Fig. S6) δ 8.27 (s, 1H), 8.16 (s, 1H), 7.14 (m, 3H), 6.83 (m, 1H), 6.75 (m, 1H), 6.12 (m, 1H), 4.92 (m, 2H), 4.63 (m, 1H), 4.51 (m, 1H), 4.35 (m, 1H), 4.21 (m, 1H), 3.44 (s, 2H), 1.62 (m, 6H), 1.18 (s, 3H), 1.11 (s, 3H). The product was confirmed by HRMS (ESI) m/z $[M+H]^+$ calculated for $C_{27}H_{29}N_7O_2Br_2$ 653.03, found 653.03.

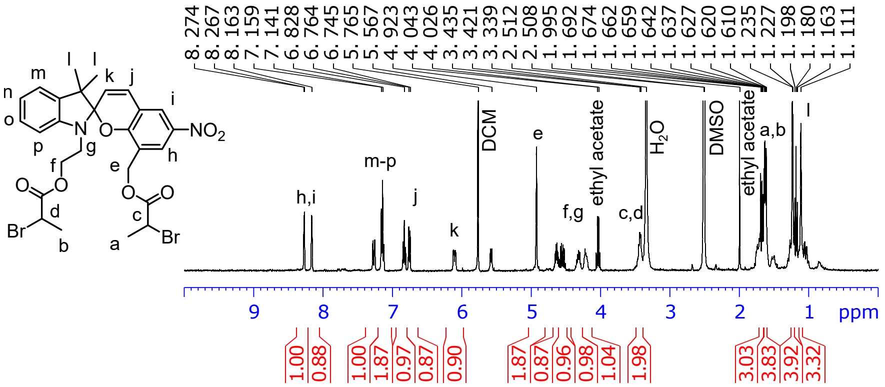

Figure S6: ^{1}H-NMR spectrum of **5** in DMSO-d$_6$ (400 MHz).

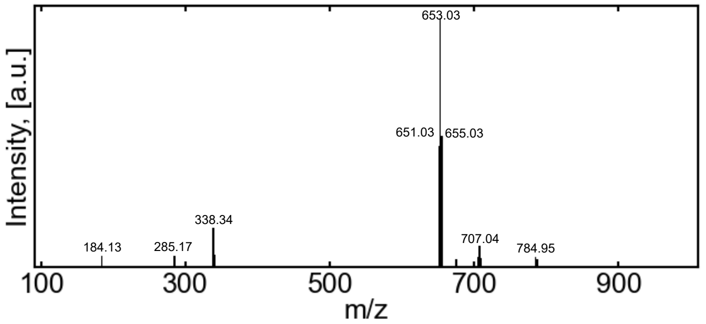

Figure S7: High-resolution mass spectrum of **5**

2 Characterization Data for MBM Triblock Copolymers

2.1 ^{1}H-NMR

^{1}H-NMR spectra of the synthesized PnBA midblock and MBM polymers are presented in Figs. S8-S10. The absence of peaks between 5 and 7 ppm indicate that all unreacted monomer was successfully removed from the polymers during the precipitation and drying steps. A trace amount of water, likely from the precipitation step, remained in the PnBA midblock, but was fully removed from the MBM triblock samples.

To determine the volume fraction of PMMA in the MBM triblock copolymers, the ^{1}H-NMR spectra of all MBM polymers were first normalized to the integral of the side chain methylene protons (a) next to the ester group in PnBA, as shown in Fig. S10. The mole fraction of PMMA was then determined from the integral of the methyl side chain protons (a′) in the PMMA repeat units. This mole fraction was further converted into the weight fraction of PMMA using the molecular weights of the nBA and MMA repeat units, and was finally converted into the resulting volume fraction f_{PMMA} using the literature densities of PnBA and PMMA (1.06 g/cm^3 and 1.18 g/cm^3, respectively[S5]).

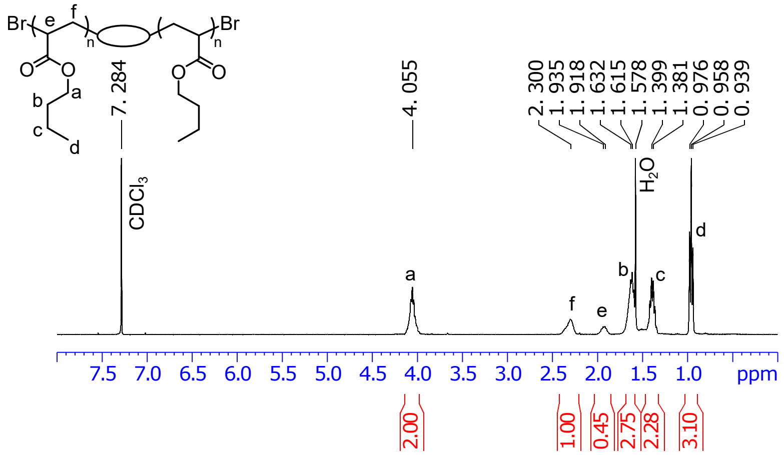

Figure S8: ^{1}H-NMR spectrum of PnBA-SP-PnBA macroinitiator in $CDCl_3$ (400 MHz).

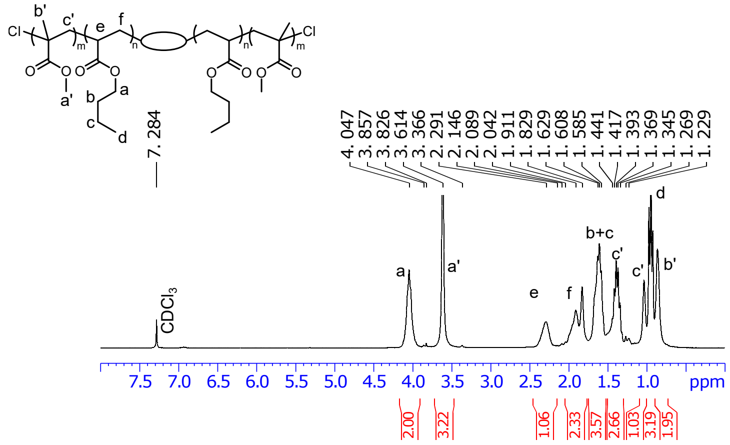

Figure S9: Representative ^{1}H-NMR spectrum of MBM in $CDCl_3$ (400 MHz).

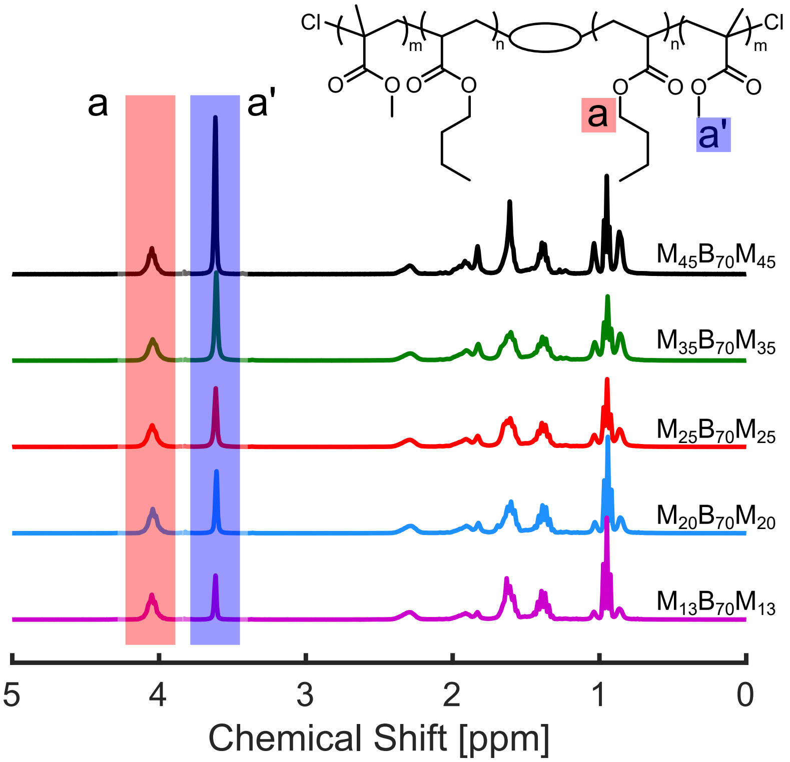

Figure S10: ^{1}H-NMR spectra of all MBM triblock copolymers in $CDCl_3$. Spectra are normalized to the 2H of the PnBA sidechain peak (a) near 4.0 ppm. Highlighted regions indicate the peaks used to determine the volume fraction of PMMA in the triblock copolymers. All spectra were obtained at 400 MHz.

2.2 SEC

SEC traces of the PnBA midblock and all five MBM triblock copolymers are shown in Fig. S11. As seen in this figure, the SEC traces shifted towards earlier elution times with in-

creasing PMMA block length, consistent with the expected increase in the molecular weight of the MBM triblock copolymer with increasing PMMA content. For the PnBA macroinitiator and the four lower molecular weight MBM triblock copolymers, a shoulder peak at high molecular weight was observed, suggesting some bimolecular chain termination. The highest molecular weight triblock copolymer, $M_{45}B_{70}M_{45}$, also exhibited a shoulder peak on the lower molecular weight side of the trace, indicating the formation of some dead polymer chains during propagation.

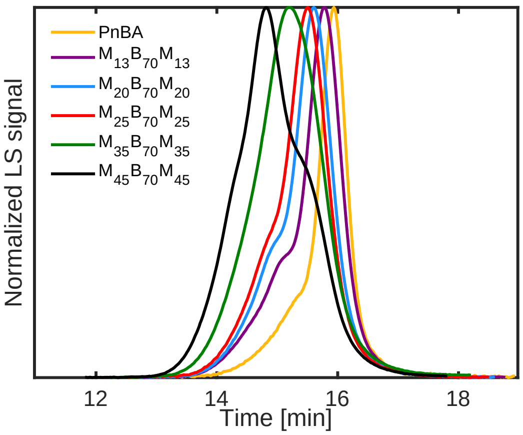

Figure S11: SEC traces of synthesized PnBA and MBM polymers before and after chain extension, measured in THF at a flow rate of 1.0 mL/min at 40 °C.

3 Tensile Measurements

3.1 Experimental Setup

Tensile samples were cut from the solvent-cast and annealed polymer films using a custom micro dog-bone die cutter, with dimensions shown in Fig. S12. The design of the die cutter is adapted from ASTM standard D638 type IV.[S6] The resulting tensile samples were then loaded onto a custom-built tensile tester, shown in Fig. S13, to perform simultaneous tensile and optical measurements. The engineering strain was calculated at each point in the measurement by dividing the displacement of the two translation stages by the initial length of the gauge section of the dog-bone (15 mm). The engineering stress was calculated at each point in the measurement by dividing the tensile force, measured by the load cell attached to the sample grips, by the initial cross-sectional area of the sample (calculated from the initial gauge width, 3 mm, and the initial gauge thickness, measured using a caliper before the tensile measurement). The absorbance of the tensile sample during deformation was recorded using a monochromatic camera, using the intensity of the light pad behind the sample as the 100% transmittance reference, and was then normalized to obtain the relative activation as described in the main text.

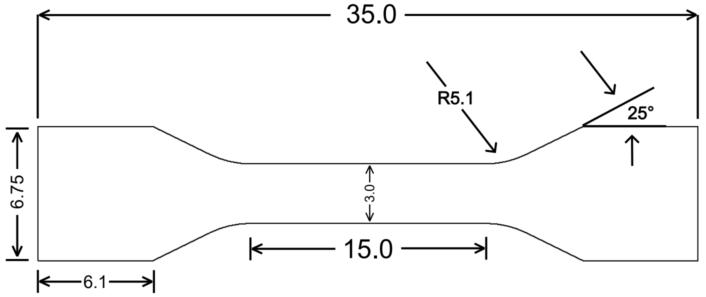

Figure S12: Custom micro dog-bone specimen for tensile test. Dimensions in mm.

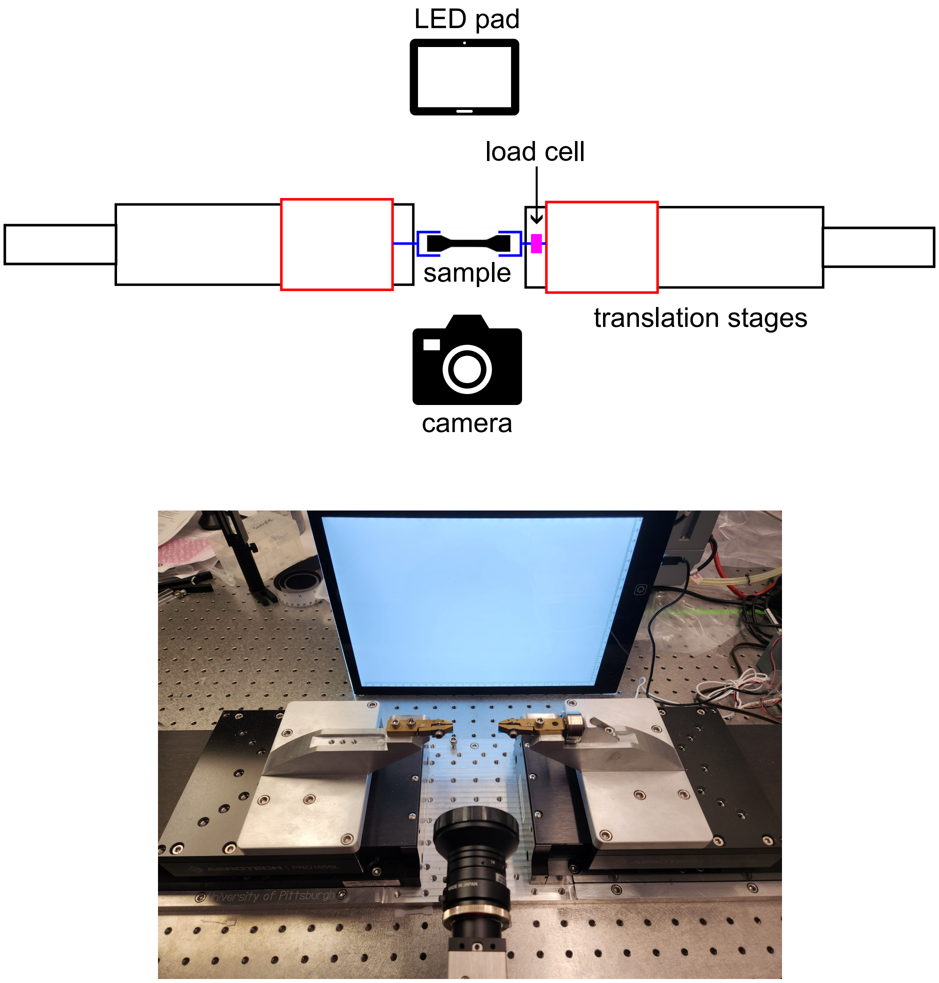

Figure S13: A schematic diagram (top) and photograph (bottom) of the custom-built tensile tester used in this work.

3.2 Determination of the Extinction Coefficient of Activated Spiropyran

As described in the main text, the fraction of spiropyran mechanophores activated during tensile deformation was determined by measuring the sample's absorbance at each timepoint in the tensile experiment, using Beer's Law to calculate the corresponding concentration of activated mechanophores, and dividing by the total concentration of spiropyran units present in the sample. Beer's Law states that the total absorbance, A, of a sample is related to the sample thickness (l), the concentration of chromophores (c), and extinction coefficient of the

chromophores (ϵ) by

$$A = \epsilon c l$$

Calculating the concentration of activated mechanophores, c, thus requires knowledge of both the sample thickness (see Section 3.3) and the extinction coefficient of the activated mechanophores.

To determine the extinction coefficient of the activated mechanophores, a calibration curve was constructed as follows. First, a small slice of the $M_{13}B_{70}M_{13}$ sample was placed between two glass slides (12-550-A3, Fisher Scientific), which were clamped together using binder clips. The thickness of the sample was measured using a micrometer, after which the sample was irradiated under a green flashlight for ~1 h before being placed between the grips of the tensile tester. The sample was imaged using the same camera, filter, and illumination source as used in the tensile experimets, and was then irradiated for ~10 mins using a UV gel nail polish dryer (DR-301C, MelodySusie, 365 nm) to drive isomerization of the spiropyran mechanophores to their ring-open form. The sample was then imaged again immediately after the UV lamp was removed. The absorbance of the activated sample was finally calculated from the intensities of the sample before and after UV irradiation.

This measurement was repeated with samples of different thicknesses, as summarized in Fig. S14. As expected, the absorbance increased with sample thickness. The data was then fit to

$$A = \epsilon \frac{\rho}{M_n} x \tag{1}$$

where A is the absorbance, x is the sample thickness, M_n is the number average molecular weight of the polymer (97 kg/mol for $M_{13}B_{70}M_{13}$), and ρ is the density of the polymer (1.085 g/cm^3 for $M_{13}B_{70}M_{13}$). The resulting molar extinction coefficient was found to be 2.0×10^4 M^{-1} cm^{-1}.

We note that this calculation, and use of this value to calculate percent activation, relies on several assumptions, namely that (1) all of the mechanophores are converted to the

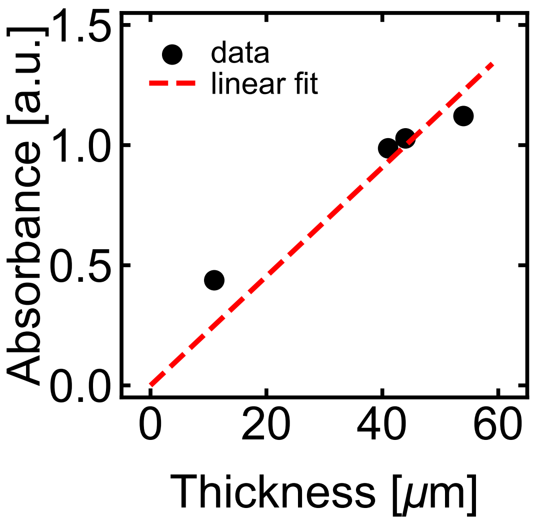

Figure S14: Absorbance of $M_{13}B_{70}M_{13}$ samples of different thicknesses after activation of the spiropyran mechanophores using UV irradiation.

ring-open spiropyran form under UV irradiation, and that (2) the extinction coefficient of the UV-activated, un-strained mechanophores is the same as the extinction coefficient of the strain-activated mechanophores, which may not be the case.[S7] As such, the molar extinction coefficient reported above, and the percent activation values reported in the main text, should be considered estimates, not exact values. However, because the same molar extinction coefficient was used to analyze all samples, any deviation from the actual activation percentages will be of the same magnitude for all samples and do not affect any of the trends reported in this work.

3.3 Thickness Normalization

The instantaneous thickness of the samples during tensile deformation was determined by measuring the instantaneous sample width in each video frame (measured in pixels) and smoothing the curve over the entire video with a polynomial fit. To obtain the instantaneous sample width, the edges of the gauge section were identified as the points where the intensity started changing sharply from the background, and the sample width was calculated as the

number of pixels between the two edges. This method only reported the measured pixel values in integers and resulted in noisy width measurements; to address this problem, we performed a polynomial fit to the data, as shown in Fig. S15, and used the fitted value for each frame as the instantaneous sample width. We then assumed that the change in the thickness of the tensile sample was directly proportional to its change in width, and the instantaneous thickness was obtained by multiplying the initial thickness by the ratio of the instantaneous sample width to the initial sample width. This sample thickness was used to normalize the measured activation as described in the main text.

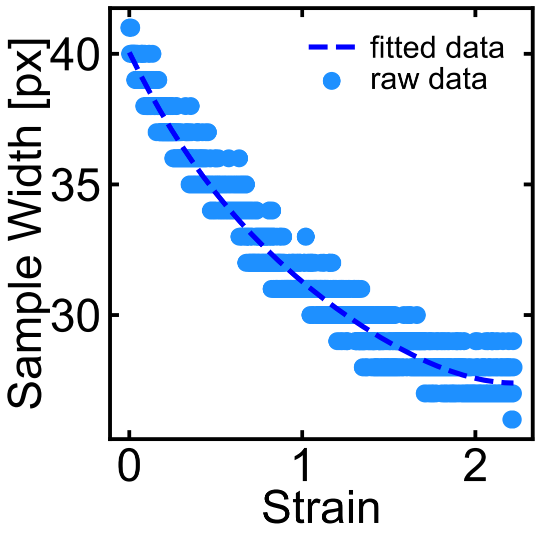

Figure S15: Representative data on sample width during tensile deformation (points), and the corresponding polynomial fit (dashed line) used to calculate instantaneous sample thickness.

3.4 Repeatability of Measurements

Stress-strain and activation-strain curves obtained from measurements on multiple samples of the same composition are shown in Figs. S16 and S17. Although the measurements stopped at different strain values, because the different sample replicates fractured at different points, the mechanical properties and the activation behaviors did not change significantly across

multiple measurements. The curves for the samples of each polymer that reached the highest strain before fracture were selected for further analysis and used in the main text.

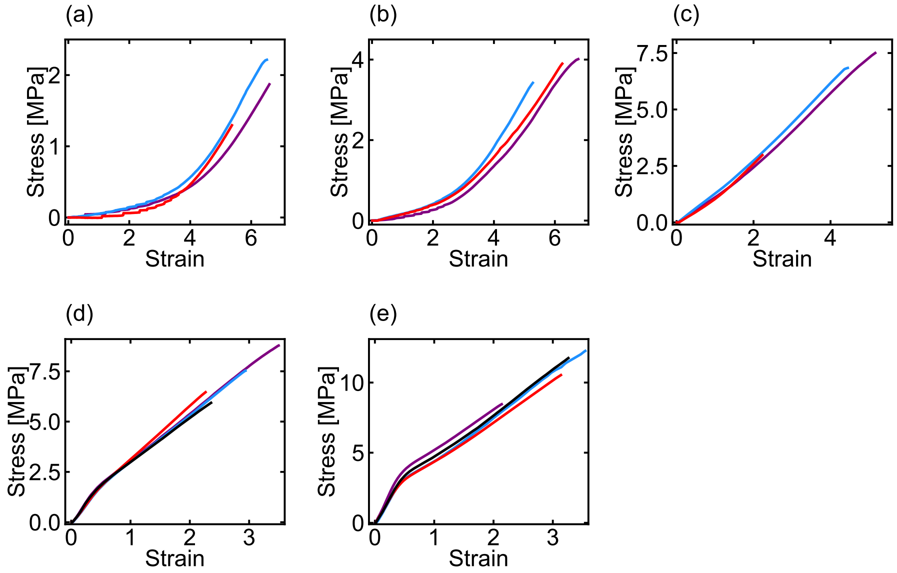

Figure S16: Stress-strain profiles of (a) $M_{13}B_{70}M_{13}$, (b) $M_{20}B_{70}M_{20}$, (c) $M_{25}B_{70}M_{25}$, (d) $M_{35}B_{70}M_{35}$, and (e) $M_{45}B_{70}M_{45}$ obtained from measurements on different dog-bone samples.

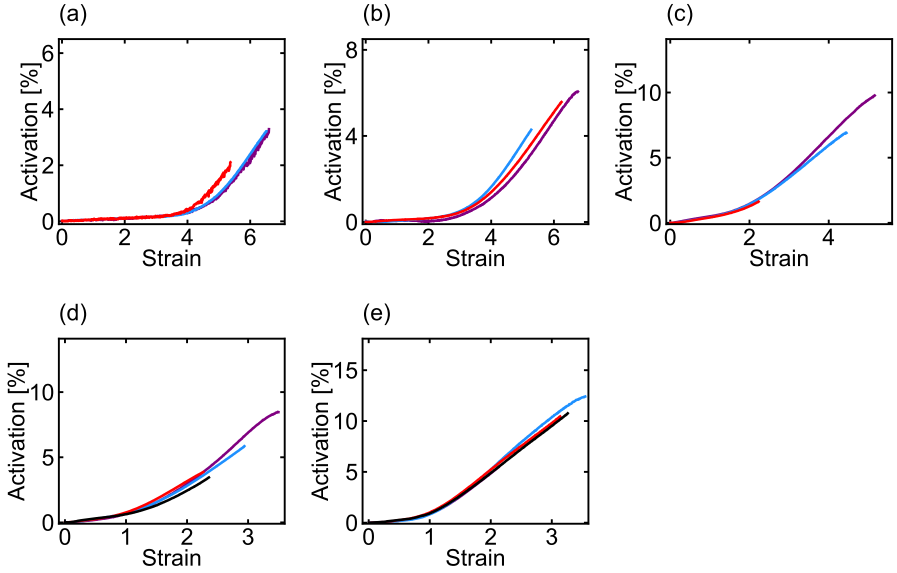

Figure S17: Activation-strain profiles of (a) $M_{13}B_{70}M_{13}$, (b) $M_{20}B_{70}M_{20}$, (c) $M_{25}B_{70}M_{25}$, (d) $M_{35}B_{70}M_{35}$, and (e) $M_{45}B_{70}M_{45}$ obtained from measurements on different dog-bone samples.

3.5 True Stress and True Strain

To facilitate comparison with our previously-reported simulation results, the stress-strain-activation curves shown in the main text were re-plotted in terms of true stress and true strain, as shown in Fig. S18. The corresponding onset points for activation and the slopes of the pre-onset and post-onset regions of each response are summarized in Figs. S19 and S20. Overall, similar trends were observed in these plots as in the data shown in terms of engineering strain and engineering stress in the main text, except for the post-onset rates, as described in the main text.

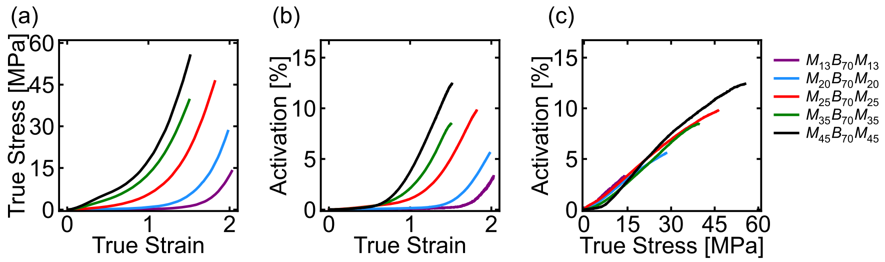

Figure S18: (a) True stress-true strain, (b) activation-true strain, and (c) activation-true stress curves for all five MBM triblock copolymers.

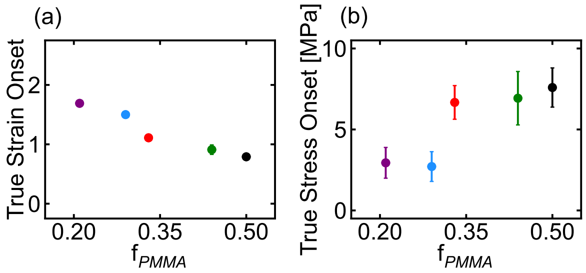

Figure S19: Onset points for activation of MBM triblock copolymers in terms of (a) true strain and (b) true stress. Error bars represent the standard deviation of 3 or 4 repeat measurements. For points that appear to be missing error bars, the error bar is smaller than the symbol size.

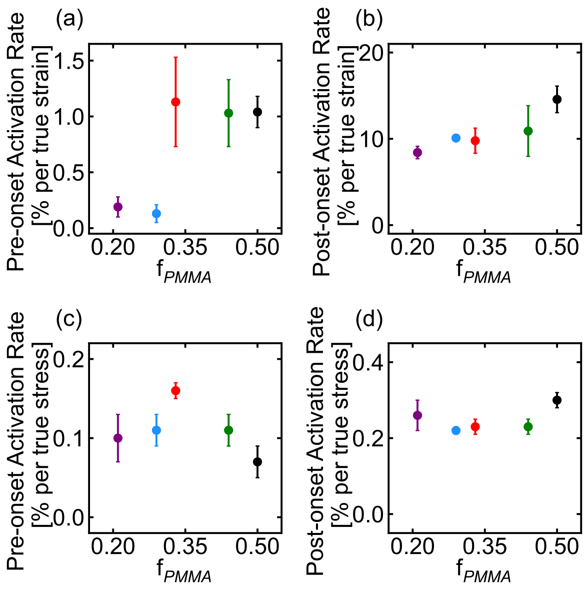

Figure S20: Activation rates before (a,c) and after (b,d) the onset point per unit (a,b) true strain and (c,d) per unit true stress. Error bars represent the standard deviation of 3 or 4 repeat measurements. For points that appear to be missing error bars, the error bar is smaller than the symbol size.

3.6 Summary of Activation Behavior

The moduli, onset points, and activation rates for all polymers reported in this work are summarized in Tables S1 and S2.

Table S1: Strain-Dependent Behavior of MBM triblock copolymers investigated in this work

**Polymer**	**Young's Modulus [MPa]**	**Strain Onset**	**Pre-onset Activation Rate [%][a]**	**Post-onset Activation Rate [%][a]**
$M_{13}B_{70}M_{13}$	0.07 ± 0.03	4.4 ± 0.3	0.06 ± 0.02	1.4 ± 0.1
$M_{20}B_{70}M_{20}$	0.20 ± 0.05	3.6 ± 0.2	0.11 ± 0.01	2.0 ± 0.1
$M_{25}B_{70}M_{25}$	1.2 ± 0.1	2.1 ± 0.2	0.7 ± 0.2	2.7 ± 0.2
$M_{35}B_{70}M_{35}$	4.1 ± 0.3	1.4 ± 0.3	0.8 ± 0.3	3.1 ± 0.6
$M_{45}B_{70}M_{45}$	7.5 ± 0.8	1.1 ± 0.1	0.8 ± 0.1	4.8 ± 0.3

[a] per unit strain

Table S2: Stress-Dependent Behavior of MBM triblock copolymers investigated in this work

**Polymer**	**Stress Onset [MPa]**	**Pre-onset Activation Rate [% MPa^{-1}]**	**Post-onset Activation Rate [% MPa^{-1}]**
$M_{13}B_{70}M_{13}$	0.8 ± 0.2	1.1 ± 0.4	2.0 ± 0.3
$M_{20}B_{70}M_{20}$	1.3 ± 0.1	0.7 ± 0.2	1.8 ± 0.1
$M_{25}B_{70}M_{25}$	2.6 ± 0.2	0.5 ± 0.1	1.5 ± 0.2
$M_{35}B_{70}M_{35}$	3.3 ± 0.2	0.16 ± 0.02	1.1 ± 0.2
$M_{45}B_{70}M_{45}$	4.5 ± 0.4	0.09 ± 0.03	1.5 ± 0.1

3.7 Onset Point Determination

The onset point was defined as the point at which lines fitted through the pre-onset (low activation) and post-onset (high activation) portions of the sample response intersected, shown in Fig. S21. The slopes of the fitted lines were reported as the pre-onset and post-onset activation rates.

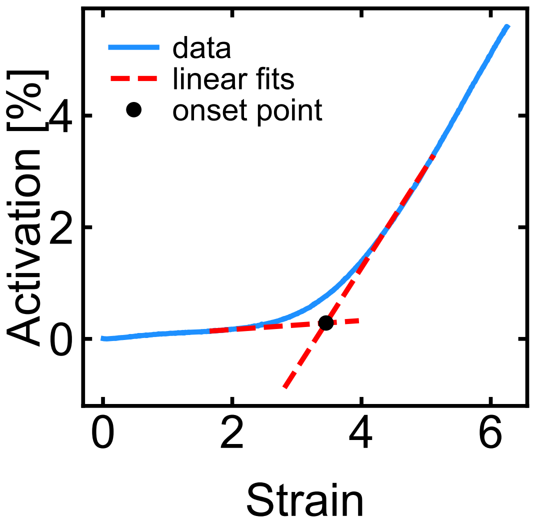

Figure S21: Representative analysis of the onset point and activation rates from an activation-strain curve, shown here for $M_{20}B_{70}M_{20}$. The blue solid line is the raw data, and the red dashed lines are the lines fitted to the pre-onset and post-onset data. The black dot indicates the intersection of the two lines, and the strain at which this point falls is defined as the onset point for activation.

3.8 Relaxation of Mechanophores during Tensile Measurements

To determine whether significant amounts of the spiropyran mechanophores revert back to their closed-ring (deactivated) form under the conditions used for the tensile tests, we strained a representative tensile sample of $M_{35}B_{70}M_{35}$ to a strain of ∼1.2, which is after its activation onset point but before its fracture point. The sample was then held at this strain for ∼900 s, comparable to the duration of a typical tensile measurement for the samples reported in this work. The resulting stress and activation as a function of time are shown in Fig. S22. The vertical dashed line represents the timestamp at which the strain reached 1.2, and the shaded area indicates the portion of the response measured after the deformation was stopped. As seen in Fig. S22(a), the stress began to relax immediately after the deformation stopped, indicating that the polymer chains were able to relax somewhat under constant strain. As seen in Fig. S22(b), however, the activation did not change significantly during this period, despite the overall relaxation of the material. This result indicates that, under tensile strain, the mechanophores are stable in the ring-open merocyanine form, and that deactivation driven by exposure to visible light from the LED light pad during tensile measurements makes a negligible contribution to the measured activation profiles.

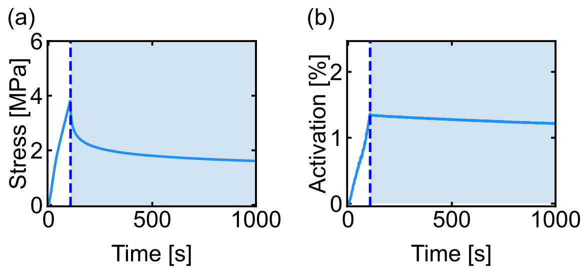

Figure S22: (a) Stress-strain curve and (b) activation-strain curve for $M_{35}B_{70}M_{35}$ strained to $\epsilon \sim 1.2$ and held at this strain for ∼900 s. The vertical dashed line indicates the time point at which the target strain was reached and the deformation was halted; to the right of this line, the strain was held constant for the remainder of the measurement.

References

(S1) Ahmed, S.; Okasha, R.; Khairou, K.; Afifi, T.; Mohamed, A.-A.; Abd-El-Aziz, A. Design of Thermochromic Polynorbornene Bearing Spiropyran Chromophore Moieties: Synthesis, Thermal Behavior and Dielectric Barrier Discharge Plasma Treatment. *Polymers* **2017**, *9*, 630, DOI: 10.3390/polym9110630.

(S2) Seo, E.; Choi, J.; Lee, B.; Son, Y.-A.; Lee, K. J. Dye Clicked Thermoplastic Polyurethane as a Generic Platform toward Chromic-Polymer Applications. *Scientific Reports* **2019**, *9*, DOI: 10.1038/s41598-019-54832-5.

(S3) Heng, S.; Mak, A. M.; Stubing, D. B.; Monro, T. M.; Abell, A. D. Dual Sensor for Cd(II) and Ca(II): Selective Nanoliter-Scale Sensing of Metal Ions. *Analytical Chemistry* **2014**, *86*, 3268–3272, DOI: 10.1021/ac500619z.

(S4) O'Bryan, G.; Wong, B. M.; McElhanon, J. R. Stress Sensing in Polycaprolactone Films via an Embedded Photochromic Compound. *ACS Applied Materials & Interfaces* **2010**, *2*, 1594–1600, DOI: 10.1021/am100050v.

(S5) Lu, W.; Goodwin, A.; Wang, Y.; Yin, P.; Wang, W.; Zhu, J.; Wu, T.; Lu, X.; Hu, B.; Hong, K.; Kang, N.-G.; Mays, J. All-acrylic superelastomers: facile synthesis and exceptional mechanical behavior. *Polymer Chemistry* **2018**, *9*, 160–168, DOI: 10.1039/c7py01518f.

(S6) Standard Test Method for Tensile Properties of Plastics. DOI: https://www.astm.org/d0638-14.html.

(S7) Kim, T. A.; Robb, M. J.; Moore, J. S.; White, S. R.; Sottos, N. R. Mechanical Reactivity of Two Different Spiropyran Mechanophores in Polydimethylsiloxane. *Macromolecules* **2018**, *51*, 9177–9183, DOI: 10.1021/acs.macromol.8b01919.
